# Native New Zealand plants with inhibitory activity towards *Mycobacterium tuberculosis*

**DOI:** 10.1186/1472-6882-10-25

**Published:** 2010-06-10

**Authors:** Emma A Earl, Mudassar Altaf, Rekha V Murikoli, Simon Swift, Ronan O'Toole

**Affiliations:** 1 School of Biological Sciences, Victoria University of Wellington, Wellington 6140, New Zealand; 2 Department of Molecular Medicine & Pathology, School of Medical Sciences, University of Auckland, Private Bag 92019, Auckland 1142, New Zealand

## Abstract

**Background:**

Plants have long been investigated as a source of antibiotics and other bioactives for the treatment of human disease. New Zealand contains a diverse and unique flora, however, few of its endemic plants have been used to treat tuberculosis. One plant, *Laurelia novae-zelandiae*, was reportedly used by indigenous Maori for the treatment of tubercular lesions.

**Methods:**

*Laurelia novae-zelandiae *and 44 other native plants were tested for direct anti-bacterial activity. Plants were extracted with different solvents and extracts screened for inhibition of the surrogate species, *Mycobacterium smegmatis*. Active plant samples were then tested for bacteriostatic activity towards *M. tuberculosis *and other clinically-important species.

**Results:**

Extracts of six native plants were active against *M. smegmatis*. Many of these were also inhibitory towards *M. tuberculosis *including *Laurelia novae-zelandiae *(Pukatea). *M. excelsa *(Pohutukawa) was the only plant extract tested that was active against *Staphylococcus aureus*.

**Conclusions:**

Our data provide support for the traditional use of Pukatea in treating tuberculosis. In addition, our analyses indicate that other native plant species possess antibiotic activity.

## Background

Tuberculosis (TB) is the leading cause of death due to a single infectious organism [1]. In 2007, 1.78 million people died from the disease and an estimated 9.27 million new cases were recorded worldwide [2]. TB requires a lengthy treatment period of six months with the first-line drugs rifampicin, isoniazid, ethambutol and pyrazinamide [3]. The availability of new drugs that shortened the course of chemotherapy would improve patient adherence and affordability thus, enabling more favourable treatment outcomes. In addition, alternative drugs are needed to counteract the spread of drug resistant TB which threatens global control programmes [4]. MDR-TB, resistant to rifampicin and isoniazid, now exceeds 0.5 million cases per year [2] and in some states accounts for up to 22% of TB cases [2,5]. Extensively-drug resistant (XDR) strains of *M. tuberculosis*, resistant to both first- and second-line drugs, were first reported in the United State, Latvia and South Korea in 2006 [6] but are now present in 57 countries [7].

The World Health Organisation has advised that new TB drugs are required to treat TB [8,9]. While much of the focus has been on screening libraries of synthetic compounds for mycobacterial inhibitors, plants have often provided a source of anti-bacterial compounds. For example, Aegicerin, isolated from *Clavija procera*, has been shown to have activity against *Mycobacterium tuberculosis *H37Rv and MDR-TB strains [10]. Rhodomyrtone, a compound from the Southeast Asian plant *Rhodomyrtus tomentosa *was found to have potent antibacterial activity against a number of clinically-important Gram-positive species including *Enterococcus faecalis*, *Streptococcus pneumoniae *and methicillin-resistant *Staphylococcus aureus *(MRSA) [11].

In this work, solvent extracts were prepared from 45 native New Zealand plants and screened for activity using *Mycobacterium smegmatis*. Active samples were then tested against *Mycobacterium bovis *BCG, *M. tuberculosis *H37Ra, *Escherichia coli *and *Staphylococcus aureus. *A number of plants were identified as containing anti-*Mycobacterium tuberculosis *activity and their potential for generating novel compounds for the treatment of tuberculosis is discussed.

## Methods

### Bacterial strains and plasmids

The mycobacterial strains used were *Mycobacterium smegmatis *mc ^2^155, *M. bovis *BCG (Pasteur) and *M. tuberculosis *H37Ra. *M. smegmatis *and *M. bovis *BCG were labelled with GFP through the introduction of the plasmid pSHIGH+hsp60 by electroporation and selection on media containing 50 μg/ml kanamycin as previously described [12]. GFP-labelled *Staphylococcus aureus *Newman was constructed as follows: the *fdh *(formate dehydrogenase) promoter was amplified from *S. aureus *Newman using Phusion™ Flash High-Fidelity PCR Master Mix (Finnzymes, Finland) according to the manufacturers' instructions with primers GGGGACAACTTTGTATAGAAAAGTTGgaaggggtaagtgtgataagc and GGGGACTGCTTTTTTGTACAAACTTgtctttaaaataagaagttcattaattgttc, where uppercase represents *attB *sites for Gateway cloning (Invitrogen) and lower case represents *S. aureus *DNA. The 275 bp amplified product was cloned into pDONR221 using BP Clonase™ II (Invitrogen) and used to transform chemically-competent *Escherichia coli *TOP 10 (Invitrogen). The insert was sequenced to confirm an error-free amplification. Gateway cloning with LR Clonase™ II (Invitrogen) combined the *fdh *promoter with *gfp*, or *gfpluxABCDE*, and the *rrnBT1T2 *terminator in the destination vector pDEST-pUNK1 as described previously [13]. The correct plasmid constructs from bioluminescent/fluorescent LR-reaction transformants of *E. coli *TOP 10 were confirmed by restriction mapping. The plasmids generated were electroporated into *S. aureus *RN4220 as previously described [14] and then transferred to *S. aureus *Newman by bacteriophage transduction [15]. *S. aureus *strains containing pDEST-pUNK1 derivatives were selected on media containing 30 g/ml erythromycin. *Escherichia coli *DH5α was labelled with GFP using pOT11 [16].

### Plant Collection and solvent extraction

Plant samples were collected from the Karori Wildlife Sanctuary (Zealandia) and the Otari-Wilton Bush Reserve, Wellington; Kaitoke Regional Park, Upper Hutt; and the Nelson region with permission. The native New Zealand plants tested for anti-mycobacterial activity are listed in Table 1. Plant samples were collected fresh, macerated and dried in a desiccator. For each sample, approximately 1 g of dried plant tissue was placed in a 50 ml conical tube. Sterile distilled water, ethanol or methanol were added to the samples to give a final concentration of 100 mg/ml. The samples were incubated in a water bath at 55°C for 1 hour and then stored at -80°C. The incubation at 55°C was used as Māori heated plant extracts during the preparation of some traditional medicines. The resulting crude extracts were sterilized using a 0.22 μm filter prior to anti-microbial analysis.

**Table 1:** Native New Zealand plants screened for activity against *M. smegmatis*.

Plant name (common)	Part	Documented Traditional Medicinal Use
*Adiantum raddianum *(Delta maidenhair fern)	Leaf	*ni*
*Agathis australis *(Kauri)	Leaf	*ni*
*Arthropodium cirratum *(New Zealand rock lily)	Leaf	Ulcers [29]
*Asplenium bulbiferum *(Hen and chicken fern)	Leaf	*ni*
*Beilschmiedia tawa *(Tawa)	Leaf	Sore throat, colds, cough [22]
*Blechnum fluviatile *(Kiwikiwi)	Leaf	Sore tongue and mouth [30]
*Brachyglottis repanda *(Rangiora)	Leaf	Wounds, ulcers and boils [30]
*Carpodetus serratus *(Marble leaf)	Leaf	*ni*
*Clianthus puniceus *(Kakabeak)	Leaf	*ni*
*Cordyline australis *(Cabbage tree)	Leaf	Dysentery, diarrhoea and sores [30]
*Cortaderia toetoe *(Toetoe)	Shoot	Diarrhoea, bladder complaints [23,31]
*Cyathea dealbata *(Ponga)	Fronds	Treat boils [32]
*Dacrydium cupressinum *(Rimu)	Leaf	Treat sores [22]
*Dracophyllum longifolium *(Inanga)	Leaf	*ni*
*Dodonaea viscosa *(Ake ake)	Leaf	Treat sore throats and skin irritations [33]
*Dysoxylum spectabile *(Kohekohe)	Leaf	Relieve coughing, colds and fevers [33]
*Exocarpus bidwilli*	Leaf	*ni*
	Seed	*ni*
*Hebe stricta *(Koromiko)	Leaf	Skin problems, ulcers, diarrhoea, dysentery, kidney and bladder complaints [33]
	Flower	*ni*
*Hymenophyton flabellatum *(Liverwort)	Leaf	*ni*
*Knightia excelsa *(Rewarewa)	Leaf	Relieve coughing [22]
*Kunzea ericoides *(Kanuka)	Leaf	Relieve pain, headaches and coughs [22]
*Laurelia novae-zelandiae *(Pukatea)	Inner bark	Sores, ulcers, toothache and tuberculosis [23,30,32]
	Bark	Treat sores, skin conditions, venereal disease [29]
	Leaf	Toothache [22]
*Leptospermum scoparium *(Manuka)	Leaf	Colds, pains, urinary troubles [30]
	Flower	*ni*
*Lophomyrtus bullata *(Ramarama)	Leaf	Treat bruises [22]
*Macropiper excelsum *(Kawakawa)	Leaf	Rheumatism, arthritis, diuretic, bruises and chest difficulties [32,34]
*Melicytos ramiflorus *(Mahoe)	Leaf	Treat rheumatism and stomach wounds [30]
*Meryta sinclairii *(Puka)	Leaf	*ni*
*Metrosideros excelsa *(Pohutukawa)	Leaf	*ni*
	Flower	Sore throats [23,30]
*Metrosideros robusta *(Northern Rata)	Leaf	*ni*
*Metrosideros umbellata *(Southern Rata)	Leaf	*ni*
*Myoporum laetum *(Ngaio)	Leaf	Bruises and infected wounds [30]
	Leaf	Bruises and wounds
*Nothofagus fusca *(Red beech)	Leaf	*ni*
*Paesia scaberula *(Lace fern)	Leaf	*ni*
*Phormium tenax *(New Zealand flax)	Leaf	Treat cuts and constipation [28]
*Pittosporum tenuifolium *(Kohuhu)	Leaf	Eczema and other skin diseases, fever and chills [22]
*Plagiochila stephensoniana *(Liverwort)	Leaf	*ni*
*Pseudopanax arboreus *(Five finger)	Leaf	*ni*
*Pseudopanax crassifolius *(Lancewood)	Leaf	*ni*
*Pteridium esculentum *(Bracken)	Leaf	Antiseptic, treats sores [33]
*Rubus cissoides *(Bush lawyer)	Leaf	Chest congestion, coughs and sore throats [30]
*Schefflera digitata *(Seven finger)	Leaf	*ni*
*Sophora microphylla *(Kowhai)	Leaf	Wounds, back, abdominal and internal pains [32]
*Urtica ferox *(Stinging nettle)	Leaf	*ni*
*Vitex lucens *(Puriri)	Leaf	Backache, ulcers, and sore throats [30]
*Weinmannia racemosa *(Kamahi)	Leaf	*ni *

*ni *= no information available.

### Screening plant extracts for bacteriostatic activity towards M. smegmatis

Plant extracts were tested for bacteriostatic activity in a 96 well-plate format assay using *M. smegmatis *mc ^2^155/pSHIGH+hsp60 as previously described [12]. Optical Density (OD) and GFP fluorescence were used to detect growth inhibition. *M. smegmatis *was cultured for 24 hours in Luria Broth (LB) supplemented with D-arabinose (1 mg/ml), Tween 80 (0.1% v/v) and kanamycin (50 μg/ml). Sterile distilled water (150 μL) was added to the outer lanes of the 96-well microtiter plates to minimise evaporation. Liquid media (50 μL) was added to the remaining wells. The effect of the solvents used on the growth of *M. smegmatis *was tested. Inhibition of *M. smegmatis *growth was observed at concentrations of ethanol and methanol greater than 5% (v/v). Plant extracts were added to columns 2 and 3 of the microtitre plates with a starting concentration of 2 mg/ml corresponding to a solvent concentration of 2% (v/v). A two-fold serial dilution was then performed on each extract starting at column 3. *M. smegmatis *cells were added to rows B-D at a final OD at 600 nm (OD _600_) of 0.2. Rows E-G contained media and extract alone, to measure any background optical density or fluorescence associated with the extract. A number of controls were added to ensure assay reliability, including the use of standard anti-tubercular drugs, rifampicin and streptomycin, as positive controls, each starting at a concentration of 100 μM. All extracts and controls were tested in triplicate. Plates were sealed and incubated at 37°C with 200 rpm shaking for 96 hours, after which the plates were read for OD and GFP fluorescence. For active extracts, dose response experiments were performed to validate activity versus *M. smegmatis*.

### Bacteriostatic assays for M. bovis, M. tuberculosis, S. aureus and E. coli

*M. bovis *BCG and M. *tuberculosis *H37Ra cultures were grown in Middlebrook 7H9 broth supplemented with 10% (v/v) OADC (0.06% oleic acid, 5% BSA, 2% Dextrose, 0.85% NaCl), glycerol (0.5% v/v) and Tween 80 (0.05% v/v). A bacteriostatic assay was set up as previously described [12], with cells added to give a final OD _600 _of 0.05 _. _All extracts and controls were tested in triplicate. Plates were sealed and incubated at 37°C with 200 rpm shaking for 14 days before the GFP fluorescence and/or OD of the cultures were measured.

GFP-labelled *S. aureus *Newman was cultured in LB containing erythromycin (30 μg/ml) and xylose (0.5% w/v). GFP-labelled *E. coli *DH5α/pOT11 was cultured in LB containing chloramphenicol (25 μg/ml). IPTG (100 μg/ml) was added to induce GFP expression. The bacteriostatic assay for *S. aureus *and *E. coli *were performed as per the *M. smegmatis *bacteriostatic assay method except cultures were incubated for 24 hours at 37°C with 200 rpm shaking post addition of extracts.

### Determination of inhibitory concentrations of plant extracts

A Perkin Elmer Envision 2102 multilabel plate reader and the Wallace Envision Manager 1.12 software program were used to measure the OD and GFP signals of the microtitre plate cultures. OD was measured at 600 nm. GFP fluorescence was detected using excitation and emission wavelengths of 485 nm and 510 nm, respectively. 12-point scans were performed on each well to minimise intra-well variation. The intrinsic absorbance and fluorescence readings of extracts alone were measured to account for background signal and subtracted from the readings for the test samples. Data were normalised by expressing the absorbance and fluorescence values as a percentage of a no-drug negative control. Dose-response curves were plotted using SigmaPlot (version 10.0) and minimum inhibitory concentration (MIC) and 50% inhibitory concentration (IC _50_) values were calculated.

## Results

### Activity of plant extracts towards M. smegmatis

45 plants native to New Zealand (Table 1) were extracted with water, ethanol and methanol and the extracts were tested for their ability to inhibit the growth of the fast-growing species, *M. smegmatis*. Extracts from 6 plants species, *Laurelia novae-zelandiae*, *Lophomyrtus bullata*, *Metrosideros excelsa*, *Myoporum laetum*, *Pittosporum tenuifolium *and *Pseudopanax crassifolius *showed inhibition towards *M. smegmatis *(Table 2). Dose-response experiments were performed on the active extracts and their MIC and IC _50 _values were determined. The most active extract was derived from *L. novae-zelandiae *(Pukatea). The bark of *L. novae-zelandiae *generated an IC _50 _value of 0.02 mg/ml, respectively while the cambium had an IC _50 _of 0.25 mg/ml (Table 3). Significant activity was also observed with respect to the leaf, IC _50 _of 0.11 mg/ml, and flower, IC _50 _of 0.41 mg/ml, of *M. excelsa*. The leaf of *P. tenuifolium *was less active with an IC _50 _value of 0.78 mg/ml (Table 3).

**Table 2:** Anti-bacterial activity of plant extracts towards *M. smegmatis *mc^2^155.

Plant species	Common Name	Part	Sample ocation	Extract		
				
				Aqueous	Ethanol	Methanol
*Laurelia novae-zelandiae*	Pukatea	Bark	Otari-Wilton	+	-	-
		Cambium	Otari-Wilton	-	-	+
*Lophomyrtus bullata*	Ramarama	Leaf	Otari-Wilton	-	-	+
*Metrosideros excelsa*	Pohutukawa	Leaf	Ngaio	-	-	+
		Flower	Ngaio	-	+	-
*Myoporum laetum*	Ngaio	Leaf	Ngaio	+	-	-
*Pittosporum tenuifolium*	Kohuhu	Leaf	Ngaio	-	+	-
*Pseudopanax crassifolius*	Lancewood	Leaf	Otari-Wilton	-	-	+

'+' indicates ≥ 90% inhibition of *M. smegmatis *growth at a concentration of 2 mg/ml or lower.

**Table 3:** MIC and IC_50 _values of active plant extracts with respect to *M. smegmatis *and clinically-relevant bacterial species.

Plant	Part		*M. smegmatis*			*M. bovis*			*M. tuberculosis*			*S. aureus*	
			**mg/ml**			**mg/ml**			**mg/ml**			**mg/ml**	
			**MIC**	**IC**_**50**_		**MIC**	**IC**_**50**_		**MIC**	**IC**_**50**_		**MIC**	**IC**_**50**_

*L. novae-zelandiae*	Bark		0.04	0.02 ± 0.01		1.50	0.54 ± 0.03		4.16	2.39 ± 0.33		> 10	> 10
	Cambium		0.50	0.25 ± 0.01		2.50	1.63 ± 0.21		3.75	1.86 ± 0.22		> 10	> 10
*L. bullata*	Leaf		1.00	0.31 ± 0.15		3.12	2.59 ± 0.43		6.51	5.59 ± 0.32		> 10	> 10
*M. excelsa*	Leaf		0.63	0.11 ± 0.06		8.35	6.95 ± 3.07		> 10	4.83 ± 0.43		2.20	1.17 ± 0.39
	Flower		0.63	0.41 ± 0.06		3.13	1.12 ± 0.12		4.18	2.19 ± 0.76		> 10	> 10
*M. laetum*	Leaf		0.63	0.34 ± 0.02		> 10	> 10		> 10	> 10		> 10	> 10
*P. tenuifolium*	Leaf		2.00	0.78 ± 0.34		> 10	5.37 ± 1.35		1.25	0.51 ± 0.11		> 10	> 10
*P. crassifolius*	Leaf		0.63	0.31 ± 0.01		6.25	4.08 ± 1.76		3.58	1.74 ± 0.18		> 10	> 10

MIC, minimum inhibitory concentration. IC_50_, 50% inhibitory concentration.

### Antibacterial activity of plant extracts towards clinically-relevant species

The extracts of *L. novae-zelandiae*, *L. bullata*, *M. excelsa*, *M. laetum*, *P. tenuifolium *and *P. crassifolius *were tested against *M. bovis *BCG and *M. tuberculosis *H37Ra. The leaf of *P. tenuifolium *was the most active extract with respect to *M. tuberculosis *with an IC _50 _of 0.51 mg/ml (Table 3). The bark taken from *L. novae-zelandiae *had an IC _50 _of 0.54 mg/ml against *M. bovis *and an IC _50 _of 2.39 mg/ml against *M. tuberculosis *(Table 3). The cambium of *L. novae-zelandiae *was similarly active against both *M. bovis *and *M. tuberculosis*. The leaf extract from *M. laetum *was not active against *M. tuberculosis*. To examine the specificity of the anti-bacterial activity of the plant extracts, the extracts were also tested against *S. aureus *and* E. coli*. The leaf of *M. excelsa *which was active against *M. smegmatis *and to a lesser extent, *M. tuberculosis*, also displayed antibacterial activity towards *S. aureus *with an IC _50 _of 1.17 mg/ml. None of the extracts tested exhibited anti-bacterial activity towards *E. coli *up to the 10 mg/ml concentration tested.

## Discussion

Plants and their extracts have been used by indigenous peoples for the treatment of infectious diseases such as tuberculosis since long before the discovery of antibiotics. A recent study determined that more than 80 plant species have been used by traditional medical practitioners in Uganda to treat tuberculosis [17]. Another study characterised leaf, bark and root material from trees used as traditional anti-tubercular medicines in South Africa. Direct bacteriostatic activity towards *Mycobacterium aurum *was identified for *Acacia nilotica *and *Combretum kraussii *at concentrations ranging from 1.56 to 0.195 mg/ml [18]. A survey of the ethnobotanical literature was used to select plants used by traditional healers in Mexico to test for activity towards *M. tuberculosis*. Extracts of *Citrus aurantifolia*, *C. sinensis *and *Olea europaea *were found to be active against both drug-susceptible and drug-resistant strains of virulent *M. tuberculosis *with minimum inhibitory concentrations of between 0.1 and 0.025 mg/ml [19]. Although, the New Zealand shrub *Lophomyrtus bullata *(Ramarama) has been identified as having activity towards *Bacillus subtilis *[20], to current knowledge, Pukatea or other New Zealand plants have not been assayed for activity against mycobacteria [21].

**Figure 1:**
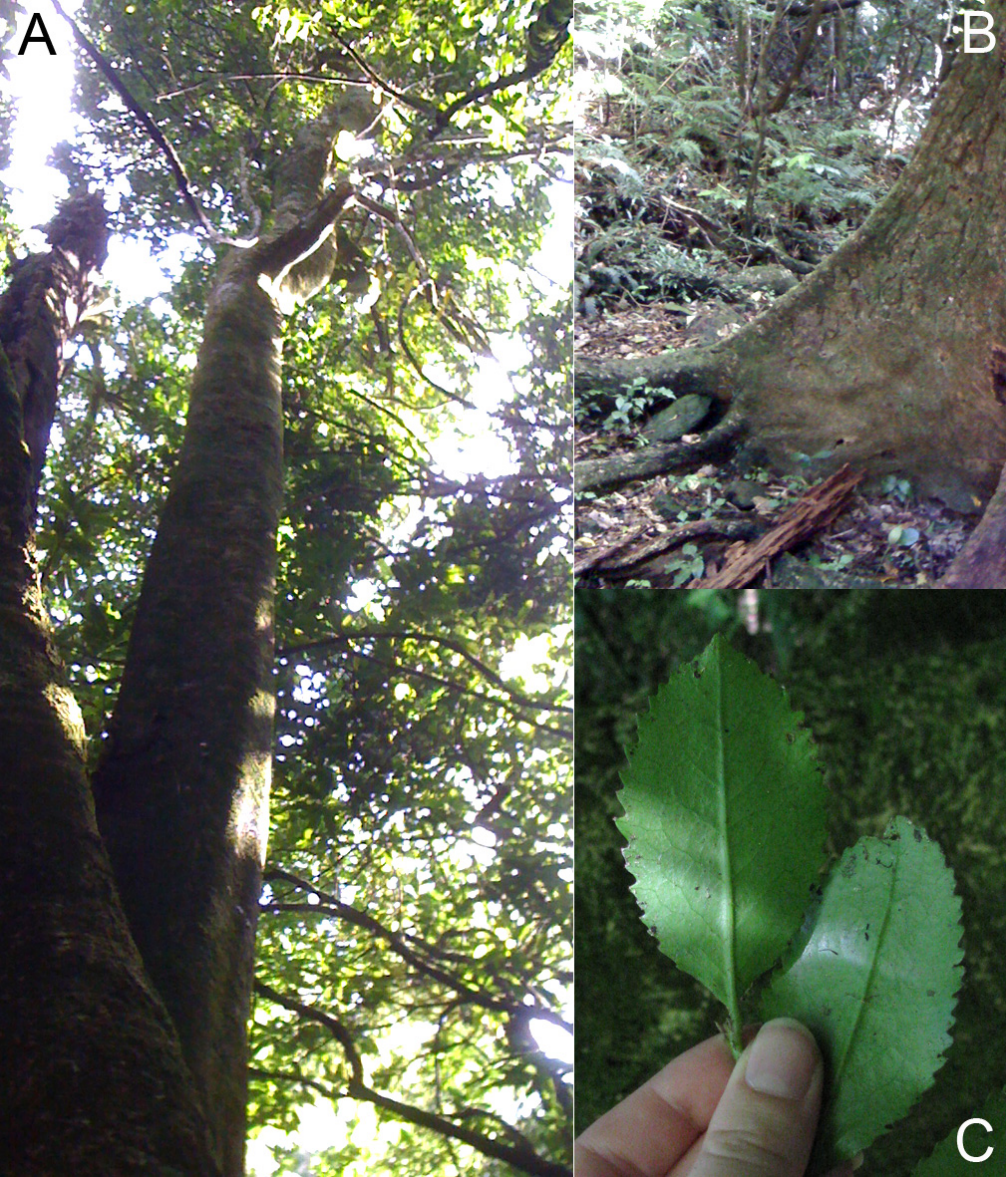
**Images of *Laurelia novae-zelandiae *(Pukatea) specimen located at Otari-Wilton Bush, Wellington**. A, branchless trunk and canopy of Pukatea. B, plank buttress of Pukatea. C, examples of "toothed" leaves of Pukatea.

In New Zealand, the bark of *Laurelia novae-zelandiae *(Pukatea) (Figure 1A) was used by Māori in a number of medicinal remedies but it was especially noted for its use against tubercular lesions [22,23]. The pulp of the cambium was boiled in water and the resulting liquid used for treating tubercular ulcers. The timber of Pukatea was also used by Māori to create figureheads for canoes [24]. Pukatea is generally found in lowland forests in the North Island and the northern areas of the South Island [25]. It can grow up to 35 metres and produces plank buttresses (Figure 1B) to support the tree's growth in swamp or shallow-soil areas [25]. Pukatea has so-called 'toothed' leaves (Figure 1C) and produces small flowers [25].

In this work, we characterised Pukatea and 44 other native plants for activity towards *M. smegmatis*. Extracts from six New Zealand plants were found to have activity against *M. smegmatis*. Bark and cambium samples from Pukatea were the most active against *M. smegmatis *(Table 3). Furthermore, the bark and cambium extracts were also active against *M. tuberculosis*. The direct anti-mycobacterial activity of the Pukatea extract therefore correlates with the use of this plant by Māori to treat tubercular lesions. This strengthens the possibility that the bark of Pukatea contains previously-unknown anti-tubercular compound(s). In Māori medicine, *M. excelsa *was used in the treatment of sore throats. Although the traditional use of *M. excelsa *does not include the treatment of tuberculosis, extracts from *M. excelsa *were nevertheless active against *M. smegmatis *and *M. tuberculosis*. Similarly, the leaf of *P. tenuifolium*, used by Māori to treat skin infections, was active against *M. smegmatis *and *M. tuberculosis*. The anti-mycobacterial activities that were detected for *M. excelsa *and *P. tenuifolium *extracts could relate to their traditional use in anti-infective therapy. Of the extracts shown to have anti-mycobacterial activity, only the *M. excelsa *leaf extract displayed activity against *S. aureus*. Interestingly, none of the plant species tested exhibited antibacterial activity towards *E. coli*. It should be noted that the MIC and IC _50 _values of the extracts in the different mycobacteria (Table 3) will be subject to variation due to species differences, the type of growth medium used, and the assay incubation time.

There is growing interest in identifying the compounds responsible for the anti-mycobacterial activity of traditional medicines and developing them as potential new tuberculosis drugs. Although the chemical basis for the anti-tubercular activity of Pukatea needs to be elucidated, earlier chemical studies identified a novel alkaloid, pukateine, from the bark of Pukatea. Pukateine is an analgesic which acts as a D _2 _dopamine receptor agonist and α1 adrenergic receptor antagonist [26,27]. A number of the other plant extracts found to have activity against mycobacteria have also been analysed chemically. The flower of *M. excelsa *contains ellagic acid which is a polyphenol antioxidant [28]. Bullatenone, discovered in *L. bullata *(Ramarama), is an antiseptic [28]. Chemical derivatives of bullatenone have been found to have anti-microbial properties against *Bacillus subtilis *[20]. Ngaione, a hepatotoxic sesquiterpene, found in the leaves of *M. laetum *(Ngaio) has been used in the treatment of athletes foot [28]. It is possible that compounds such as pukateine, ellagic acid, bullatenone or ngaione may contribute to the anti-bacterial activity of their plant extracts. These compounds offer a starting point for future studies to characterise the chemical basis of the anti-bacterial activity of *L. novae-zelandiae, M. excelsa*, *L. bullata *and *M.laetum*. They may also assist in identifying ways to improve the efficiency of the extraction of anti-mycobacterial activity from the plant samples and hence, provide extracts with lower MIC values towards *M. tuberculosis*. Such extracts will be integral to any bioassay-guided purification of active compounds.

## Conclusions

It is estimated that less than 10% of the Earth's higher plant species have been analysed for any kind of bioactivity [18]. There is a need for more research to be conducted on plants that have been traditionally used by indigenous peoples to treat tuberculosis, for the identification of new anti-tubercular drugs. New Zealand has a diverse flora which potentially offers many unique bioactive compounds. Drawing on knowledge from traditional medicine, we have identified a number of native plants which contain activity against *M. tuberculosis*. Determining the chemical species responsible for this activity will be the subject of further studies.

## Competing interests

The authors declare that they have no competing interests.

## Authors' contributions

EAE collected the plant samples, performed the solvent extractions and antibacterial assays with *M. smegmatis*, *E. coli *and *S. aureus*. MA perfomed the antibacterial assays in the slow-growing mycobacteria, *M. bovis *BCG and *M. tuberculosis *H37Ra. RVM assisted in testing the potency of the plant extracts. SS constructed the GFP-labelled *S. aureus*. RO'T conceived and funded the study, and planned and supervised the experimental work. All authors have read and approved the final manuscript.

## Pre-publication history

The pre-publication history for this paper can be accessed here:

http://www.biomedcentral.com/1472-6882/10/25/prepub
